# Efficacy and Safety of Ozanimod in Ulcerative Colitis: A Real-World Pan-Hellenic Study

**DOI:** 10.3390/medicina62091711

**Published:** 2026-09-06

**Authors:** Pavlos Pardalis, Alexandros Ioannou, Theodoros Delidis, Georgios Kokkotis, Georgios Leonidakis, Georgios Axiaris, Konstantinos Argyriou, Anthia Gatopoulou, Olga Giouleme, Nikolaos Kalakos, Pantelis Karatzas, Ioanna Nefeli Mastorogianni, Andreas Kapsoritakis, Efrosini Laoudi, Panagiotis Markopoulos, Konstantinos Katsanos, Georgios Michalopoulos, Konstantinos Mousourakis, Georgios Bamias, Georgios Papatheodoridis, Ioanna Papatzelou, Konstantinos Soufleris, Maria Tzouvala, Eftychia Tsironi, Ioannis Psaroudakis, Angeliki Theodoropoulou, Alexandra Varka, Nikos Viazis, Eirini Zacharopoulou, Konstantinos Karmiris, Ioannis Koutroubakis, Spyridon Michopoulos, Evanthia Zampeli

**Affiliations:** 1 Department of Gastroenterology, “Alexandra” General Hospital of Athens, 115 28 Athina, Greece; 2 Gastrointestinal Unit, 3rd Academic Department of Internal Medicine, University of Athens-Sotiria Hospital, 115 27 Athens, Greece; 3 Department of Gastroenterology, Theagenio Cancer Hospital of Thessaloniki, 546 39 Thessaloniki, Greece; 4 Department of Gastroenterology, University General Hospital of Larissa, 413 34 Larissa, Greece; 5 2nd Department of Internal Medicine, General University Hospital of Alexandroupolis, 681 00 Alexandroupolis, Greece; 6 2nd Propaedeutic Department of Internal Medicine, Aristotle University of Thessaloniki, 541 24 Thessaloniki, Greece; 7 Department of Gastroenterology, General Hospital G. Gennimatas, 115 27 Athens, Greece; 8 Department of Gastroenterology, General Hospital Laiko, 115 27 Athens, Greece; 9 Division of Gastroenterology, University Hospital and University of Ioannina, 455 00 Ioannina, Greece; 10 Department of Gastroenterology, Metaxa Cancer Hospital of Athens, 185 37 Athens, Greece; 11 Department of Gastroenterology, Evangelismos Hospital, 106 76 Athens, Greece; 12 Department of Gastroenterology, University Hospital, 715 00 Heraklion, Greece; 13 Department of Gastroenterology, General Hospital Nikaia and Pireaus “Agios Panteleimon”, 184 54 Athens, Greece; 14 Department of Gastroenterology, Venizeleio General Hospital, 714 09 Heraklion, Greece

**Keywords:** ozanimod, ulcerative colitis, inflammatory bowel disease

## Abstract

*Background and Objectives*: Ozanimod is approved for the treatment of ulcerative colitis (UC). The aim of the present study was to evaluate the clinical, biochemical, and endoscopic outcomes. *Materials and Methods*: This was a retrospective, multicenter, cohort study. Clinical, biochemical, endoscopic, and safety data were collected from medical records. Τhe primary outcome was clinical response at week 12. Secondary outcomes included clinical remission, endoscopic improvement and remission, biochemical outcomes, treatment persistence, and safety. Clinical outcomes were assessed at weeks 12 and 48, while treatment persistence was evaluated over the complete 48-week follow-up period, using Kaplan–Meier analysis. Clinical response was defined as a ≥30% reduction in partial Mayo score and remission as a partial Mayo score < 3, with no individual sub-score > 1 and a rectal bleeding sub-score of 0. Clinical response and remission were primarily assessed using an observed-case approach, with an additional sensitivity analysis classifying discontinuations due to lack of efficacy as treatment failures. *Results*: A total of 79 patients were included (62.0% male; median age 44 years, IQR 31–56). Prior biologic exposure was recorded in 29.1%, and 64.6% were previously exposed to oral corticosteroids. Among patients with available clinical assessment at week 12 (n = 62), clinical response and clinical remission were achieved in 77.4% and 71.0%, respectively. Among patients with available data at week 12 (n = 32), endoscopic improvement was observed in 65.6%, while endoscopic remission was achieved in 15.6%. At week 48, among 26 evaluable patients, clinical response and remission rates were 96.2% and 84.6%, respectively; when discontinuations due to lack of efficacy were considered treatment failures, the corresponding rates were 52.1% and 45.8%. Treatment persistence did not differ significantly according to overall prior biologic exposure, whereas previous vedolizumab exposure was associated with lower treatment persistence over the week 48 follow-up (log-rank *p* = 0.037). In multivariable Cox regression, previous vedolizumab exposure remained associated with an increased hazard of treatment discontinuation (adjusted HR 3.07, 95% CI 1.23–7.67; *p* = 0.016). Adverse events were reported in 13.9% of patients. *Conclusions*: Ozanimod demonstrated good efficacy and a favorable safety profile in patients with moderate UC.

## 1. Introduction

Ulcerative colitis (UC) is a chronic, immune-mediated inflammatory bowel disease (IBD) that is characterized by relapsing and remitting inflammation of the colonic mucosa, resulting in considerable reduction in quality of life and a large healthcare burden worldwide [1]. The treatment of UC has changed substantially over the past 20 years with the emergence of biologic and small-molecule therapies, which target important inflammatory pathways. Nevertheless, a considerable proportion of patients do not reach clinical remission or lose response over time; therefore, underscoring the need for novel effective and safe therapies [2,3].

Therapeutic options for moderate-to-severe UC include corticosteroids, immunomodulators, biologic agents directed against tumor necrosis factor-alpha (TNF-α), integrins and other interleukins, as well as oral small molecules, such as Janus kinase (JAK) inhibitors [4]. However, primary non-response, secondary loss of response, immunogenicity, and safety concerns are all limitations of current treatments. Hence, there is a constant interest in studying new drugs with adequate efficacy and acceptable safety profiles that may offer therapeutic flexibility and better patient adherence.

Ozanimod is a selective modulator of sphingosine-1-phosphate (S1P) receptor subtypes 1 and 5, which causes reversible sequestration of activated cells in lymphoid tissues and a decrease in intestinal inflammation [5]. Ozanimod is an immunomodulatory agent with selective modulation of lymphocyte trafficking. Its selectivity may reduce some of the cardiovascular and pulmonary adverse events related to other S1P receptor modulators. Ozanimod was first licensed for relapsing multiple sclerosis and was later found to be effective in individuals with moderate-to-severe UC [6].

The efficacy and safety of ozanimod in UC were mainly established in the phase 3 True North trial, which demonstrated significantly higher rates of clinical remission, clinical response, endoscopic improvement, and mucosal healing compared with placebo during induction and maintenance therapy [7]. Ozanimod has also shown sustainable efficacy in long-term extension studies with an acceptable safety profile and minimal rates of major adverse events [8]. Based on these results, it was approved for the treatment of moderately to highly active UC by the United States Food and Drug Administration (FDA) and the European Medicines Agency (EMA).

Randomized clinical trials provide strong evidence on the efficacy and safety of any new treatment; however, real-world data remain essential to evaluate their performance in routine clinical practice, where patient populations tend to be more heterogeneous and include those with comorbidities, previous biologic exposure, and concomitant corticosteroid use. Real-world studies assessing ozanimod in UC are still very scarce, especially in Southern Europe [9,10]. Furthermore, the efficacy of ozanimod in biologic-experienced patients and its safety profile in the context of routine clinical practice are areas of ongoing clinical interest.

The purpose of the present multicenter, retrospective study was, therefore, to assess the real-world efficacy and safety of ozanimod as induction therapy in patients with moderately active ulcerative colitis treated in tertiary referral centers across Greece. Clinical, biochemical and endoscopic outcomes, as well as adverse events and therapy persistence, were also studied.

## 2. Materials and Methods

### 2.1. Study Design and Population

This was a retrospective, multicenter observational cohort study including adult patients with UC, treated with ozanimod, across 14 tertiary referral centers in Greece. Patients with Crohn’s disease or those younger than 18 years were excluded. Consecutive patients fulfilling the inclusion criteria were identified through electronic medical records, and data were retrospectively collected up to December 2025. Baseline was defined as the time of ozanimod initiation, at which demographic, clinical, laboratory, and endoscopic data were recorded. Patients were included regardless of prior exposure to biologic or small-molecule therapies. In addition, a safety analysis was conducted, and adverse events occurring during follow-up were recorded. The study was conducted in accordance with the principles of the Declaration of Helsinki. All data were anonymized prior to analysis, and the investigations were conducted in accordance with the principles outlined in the Declaration of Helsinki (1975, revised in 2013), ensuring that the study adheres to both national (4624/2019 law of the Greek Personal Data Protection Authority) and international (EU GDPR 2016/679) regulations. Because of the retrospective nature of our study, which was conducted in accordance with the European guidelines, based on good clinical practice, the local ethics committee considered that formal approval was unnecessary for this kind of study and suggested obtaining verbal consent from the patients prior to initiation of data collection.

### 2.2. Data Collection

Data were retrospectively extracted from electronic medical records at each participating center. Collected variables included demographic characteristics (age, sex), disease-related characteristics (age at diagnosis, disease duration, disease extent according to the Montreal classification, and extraintestinal manifestations), smoking status, and comorbidities, including multiple sclerosis.

Treatment history was recorded, including exposure to mesalamine, corticosteroids, immunomodulators, biologic agents, and small molecules. Baseline laboratory parameters included hemoglobin, lymphocyte count, albumin, and C-reactive protein (CRP) levels. Calprotectin was not included in the analysis because it is not yet reimbursed in Greece. Disease activity was assessed using the partial Mayo score, modified Mayo score, and Mayo endoscopic sub-score (MES). Due to the retrospective design, strict assessment windows were not predefined. The assessments closest to weeks 12 and 48 were used, while baseline CRP and endoscopic data were obtained from the assessments closest to and before ozanimod initiation.

Adverse events were recorded throughout follow-up, as part of the safety assessment. All available patient data were utilized for the evaluation of treatment persistence and safety outcomes.

### 2.3. Outcomes and Definitions

The primary outcome of the study was the assessment of ozanimod effectiveness, defined by clinical response at week 12. The secondary outcomes included clinical remission at week 12, clinical response/remission at week 48, treatment persistence during the 48-week follow-up period, endoscopic outcomes at week 12 and 48, biochemical outcomes at week 12 and week 48, safety profile and adverse events (AEs). Exploratory analyses evaluated factors associated with treatment discontinuation, including the impact of previous biologic and vedolizumab exposure.

Clinical response was defined as a reduction of ≥30% in the partial Mayo score from baseline, accompanied by a rectal bleeding sub-score ≤ 1. Clinical remission was defined as a partial Mayo score < 3, with no individual sub-score > 1 and a rectal bleeding sub-score of 0. Endoscopic improvement was defined as a Mayo endoscopic sub-score ≤ 1, whereas endoscopic remission was defined as a Mayo endoscopic sub-score of 0. Biochemical response was assessed by evaluating both changes in CRP levels from baseline and the proportion of patients achieving CRP normalization (≤0.5 mg/dL). Clinical response and remission rates were primarily calculated using an observed-case approach, including patients with available clinical assessment at the respective time point. To account for potential attrition related to treatment failure, an additional sensitivity analysis was performed in which patients who discontinued ozanimod because of primary non-response or secondary loss of response before the respective assessment were classified as treatment failures. Patients who discontinued because of adverse events, were lost to follow-up, or had not yet reached the respective assessment time point were not classified as treatment failures, as lack of efficacy could not be established.

Treatment persistence was defined as the time from ozanimod initiation to permanent treatment discontinuation. Treatment discontinuation was defined as permanent cessation of ozanimod for any reason. All permanent discontinuations, irrespective of the reason, were considered events in the time-to-event analyses, while patients remaining on treatment were censored at their last available follow-up. Reasons for treatment discontinuation were categorized as primary non-response, secondary loss of response, or adverse events. No discontinuations due to surgery, pregnancy, patient preference, physician decision, or elective withdrawal after remission occurred in this cohort.

Potential factors associated with treatment discontinuation over the 48-week follow-up period were evaluated using univariable Cox proportional hazards regression analyses. Variables examined included age, sex, previous vedolizumab exposure, corticosteroid use at ozanimod initiation, disease extent, disease duration, and baseline CRP. Given the limited number of treatment-discontinuation events and the consequent risk of overfitting, the number of covariates included in the multivariable model was restricted. Age, sex, previous vedolizumab exposure and corticosteroid use at ozanimod initiation were included as clinically relevant covariates, rather than being selected only on the basis of statistical significance in univariable analysis. Hazard ratios (HRs) with 95% confidence intervals (CIs) and *p*-values were reported. Given the clinical relevance of week 12 as an assessment time point, an additional Cox regression analysis restricted to the first 12 weeks of treatment was retained as an exploratory supplementary analysis of early treatment discontinuation.

### 2.4. Statistical Analysis

Continuous variables are presented as mean ± standard deviation (SD) or median with interquartile range (IQR), as appropriate. Categorical variables are presented as frequencies and percentages.

Clinical and endoscopic outcomes at weeks 12 and 48 were evaluated in patients with available follow-up data. Changes in partial Mayo score, Mayo endoscopic sub-score, and CRP levels from baseline to follow-up were assessed using the Wilcoxon signed-rank test. Treatment persistence over time was evaluated using survival analysis methods.

Time-to-event analyses were performed using the Kaplan–Meier method to estimate treatment persistence over time. Comparisons between groups were performed using the log-rank test. Cox proportional hazards regression analysis was used to identify variables associated with treatment discontinuation. A two-sided *p*-value < 0.05 was considered statistically significant. Statistical analyses were performed using SPSS Statistics version 26 (IBM Corp., Armonk, NY, USA).

## 3. Results

### 3.1. Baseline Characteristics

**Figure 1:**
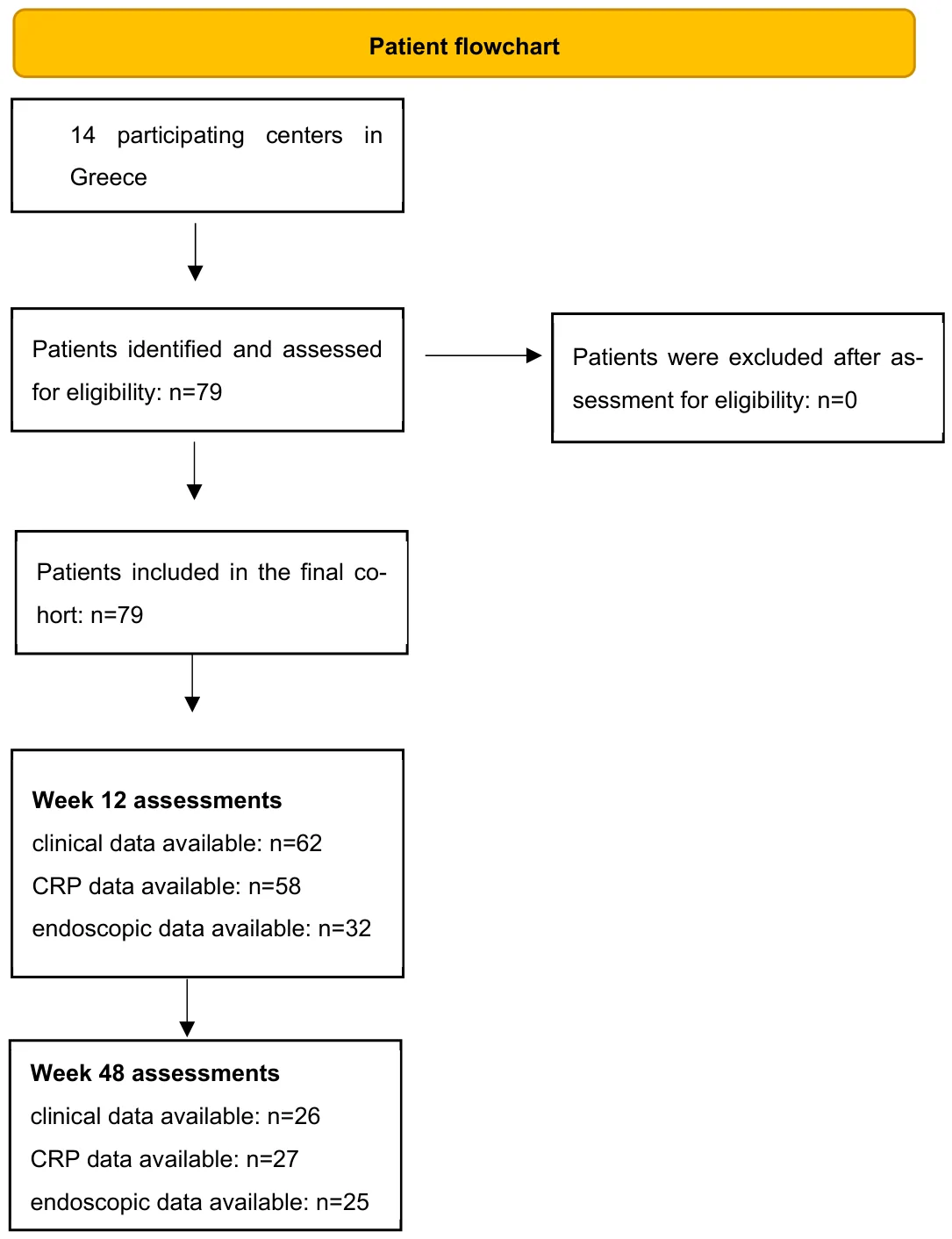
The patient flowchart.

A total of 79 patients from 14 tertiary referral centers were included in the study (patient flow chart). The median age was 44 years (IQR 31–56), 49 patients (62.0%) were male, and the median disease duration was 9 years (IQR 3–14). In total, 11 (13.9%) were current smokers. Multiple sclerosis was present in seven patients (8.9%) Figure 1.

Regarding disease extent, six patients (7.6%) had ulcerative proctitis (E1), 35 (44.3%) had left-sided colitis (E2), and 38 (48.1%) had extensive colitis (E3). Overall, 23 patients (29.1%) previously received biologic therapy. At baseline, the median CRP level was 0.4 mg/dL (IQR 0.1–1.20), the median partial Mayo score was five (IQR 3–6), and the median Mayo endoscopic sub-score was two (IQR 2–2). Additional demographic, clinical, and treatment characteristics are presented in Table 1.

### 3.2. Clinical and Endoscopic Outcomes at Week 12

**Figure 2:**
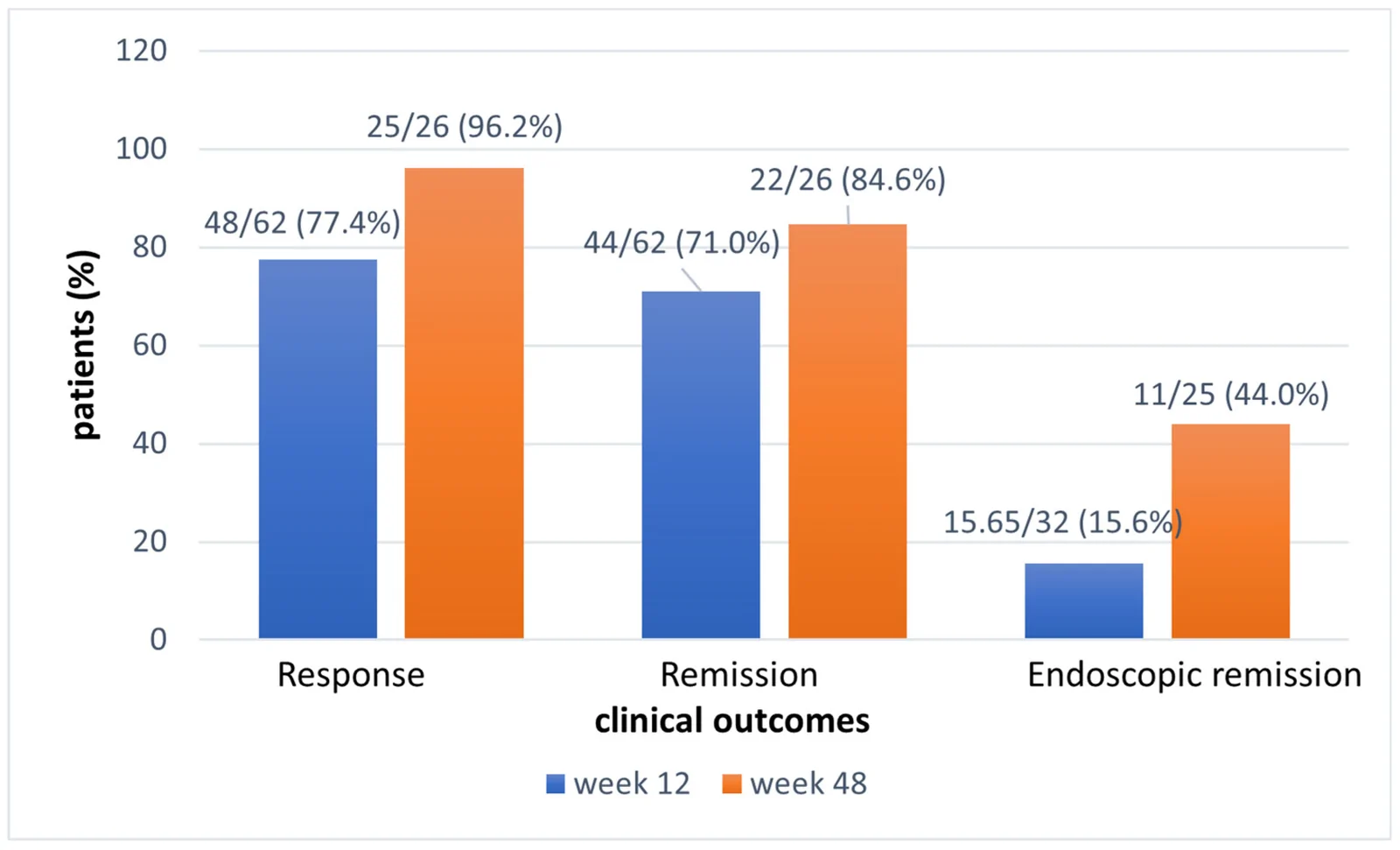
Clinical and endoscopic outcomes at weeks 12 and 48. Bars represent the proportion of patients achieving clinical response, clinical remission, and endoscopic remission, among those with available data at the time point.

Clinical outcomes at week 12 were available for 62 patients. In the observed-case analysis, clinical response was achieved in 48/62 patients (77.4%), while clinical remission was observed in 44/62 patients (71.0%) (Figure 2). In an additional sensitivity analysis, the eight patients who discontinued ozanimod before week 12 because of primary non-response were classified as treatment failures. Accordingly, clinical response and remission rates were 48/70 (68.6%) and 44/70 (62.9%), respectively. Patients who were lost to follow-up or discontinued because of adverse events were not classified as treatment failures, as lack of efficacy could not be established in these patients. A significant improvement in disease activity was observed between baseline and week 12, with median partial Mayo score—of the available data—decreasing from 4 (IQR 3–5) to 2 (IQR 0–3) (Wilcoxon signed-rank test, *p* < 0.001).

Endoscopic outcomes at week 12 were available for 32 patients. Endoscopic improvement was achieved in 21/32 patients (65.6%), while endoscopic remission was observed in 5/32 patients (15.6%) (Figure 2). Median Mayo endoscopic sub-score significantly improved from baseline to week 12, decreasing from 2 (IQR 2–2) to 1 (IQR 1–2) (*p* < 0.001).

### 3.3. Clinical and Endoscopic Outcomes at Week 48

Clinical outcomes at week 48 were available for 26 of the 79 patients. In the observed-case analysis, clinical response was achieved in 25/26 patients (96.2%), while clinical remission was achieved in 22/26 patients (84.6%) (Figure 2). In an additional sensitivity analysis, 22 patients who had discontinued ozanimod before week 48 because of primary non-response or secondary loss of response were classified as treatment failures. Accordingly, clinical response and remission rates were 25/48 (52.1%) and 22/48 (45.8%), respectively. Median partial Mayo score—of the available data—significantly improved from 4 (IQR 3–5) at baseline to 0 (IQR 0–1) at week 48 (*p* < 0.001). Among 25 patients with available endoscopic data at week 48, endoscopic improvement was achieved in 20/25 patients (80.0%), while endoscopic remission was achieved in 11/25 patients (44.0%) (Figure 2). Median Mayo endoscopic sub-score significantly improved over time (*p* < 0.001).

### 3.4. Biochemical Response

Baseline CRP values were available for 70 patients, with a median CRP level of 0.4 mg/dL (IQR 0.1–1.20). Thirty-one patients (44.2%) had elevated CRP (>0.5 mg/dL) at baseline, of whom 21 had paired week 12 data available.

In this subgroup, median CRP significantly decreased from 1.40 mg/dL (IQR 0.75–2.19) to 0.70 mg/dL (IQR 0.23–1.25) at week 12 (Wilcoxon signed-rank test, *p* < 0.01). CRP normalization (≤0.5 mg/dL) was achieved in 8/21 patients (38.1%) at week 12.

### 3.5. Missing Data Analysis

No statistically significant differences in age, baseline partial Mayo score, baseline Mayo endoscopic sub-score, baseline CRP, previous biologic exposure and corticosteroid use at ozanimod initiation were observed between patients with and without available follow-up data at weeks 12 and 48 (Supplementary Table S1). However, these baseline comparisons cannot exclude attrition bias related to post-baseline treatment outcomes, particularly when the paucity of follow-up assessment was related to treatment discontinuation, due to lack or loss of response.

### 3.6. Safety

Adverse events were reported in 11/79 patients (13.9%). Four patients discontinued ozanimod because of adverse events. These included persistent transaminasemia (n = 1), severe lymphopenia (n = 1), pulmonary embolism occurring approximately two weeks after treatment initiation (n = 1) and immune thrombocytopenia occurring approximately four weeks after treatment initiation (n = 1). A causal association with ozanimod could not be definitively established. No deaths were recorded. Non-serious adverse events included lymphopenia, not requiring treatment discontinuation (n = 3), transaminasemia (n = 3) and herpes zoster infection (n = 1). Overall, the safety profile of ozanimod was consistent with previously reported data, and no new safety signals were identified (Table 2).

### 3.7. Treatment Persistence and Survival Analysis

**Figure 3:**
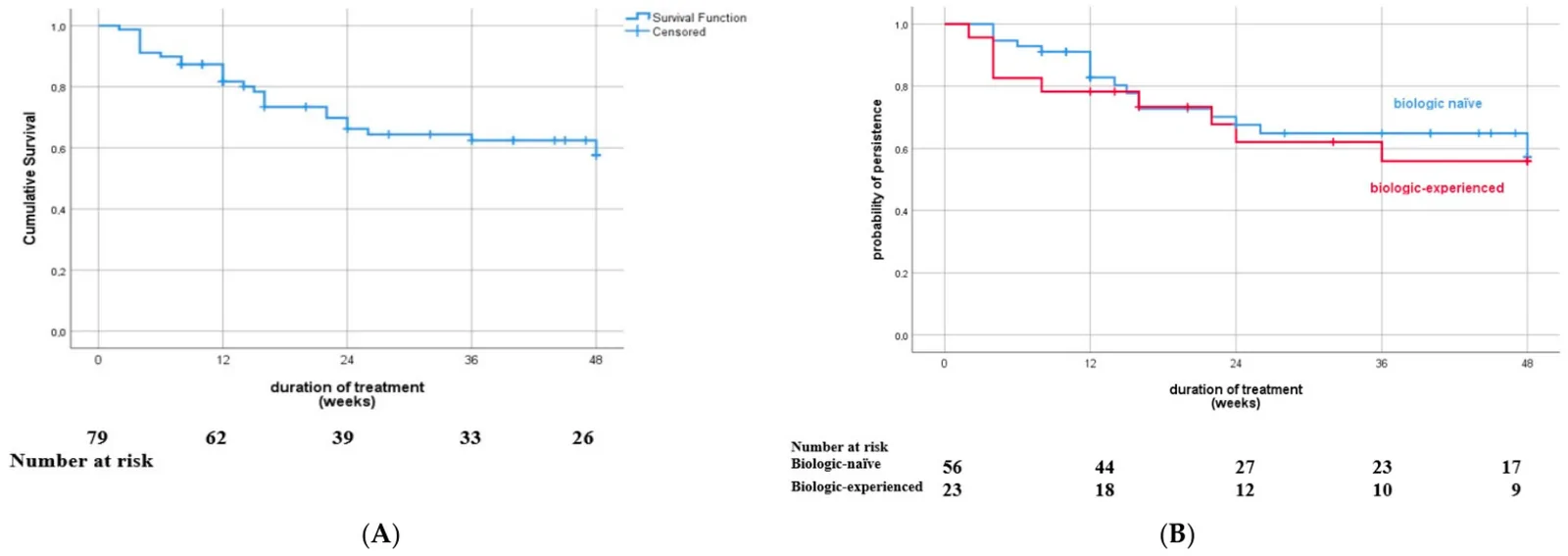
(**A**) Kaplan–Meier curve of overall ozanimod treatment persistence during the 48-week follow-up period. (**B**) Kaplan–Meier curve of treatment persistence according to prior biologic exposure during the 48-week follow-up period. Biologic-naïve (n = 56) and biologic-experienced patients (n = 23) were compared using the log-rank test (*p* = 0.729). Censored observations are indicated by tick marks. Numbers at risk are shown below the plot.

During follow-up at week 48, 28/79 patients (35.4%) discontinued treatment. Of these, 27 discontinued within the 48-week follow-up period, while one patient discontinued thereafter. Twenty-four discontinued because of lack of efficacy (primary non-response or secondary loss of response), while four discontinued because of adverse events. Reasons for treatment discontinuation according to prior biologic exposure are presented in Supplementary Table S2. Of the 28 recorded treatment discontinuations, during the available follow-up, 27 occurred within the prespecified 48-week follow-up period and were considered events in the 48-week treatment-persistence analyses; one discontinuation occurred after week 48 and was therefore not included as an event in these analyses. Kaplan–Meier analysis estimated overall treatment persistence at 81.7% at week 12 and 57.6% at week 48 (Figure 3A). We also compared drug discontinuation in biologic-naïve and biologic-experienced patients over a 48-week follow-up period. Treatment persistence did not differ significantly between the two groups (log-rank *p* = 0.729). Kaplan–Meier estimates of treatment persistence at weeks 12 and 48 were 82.8% and 57.2% in biologic-naïve patients and 78.3% and 55.9% in biologic-experienced patients, respectively (Figure 3B).

**Figure 4:**
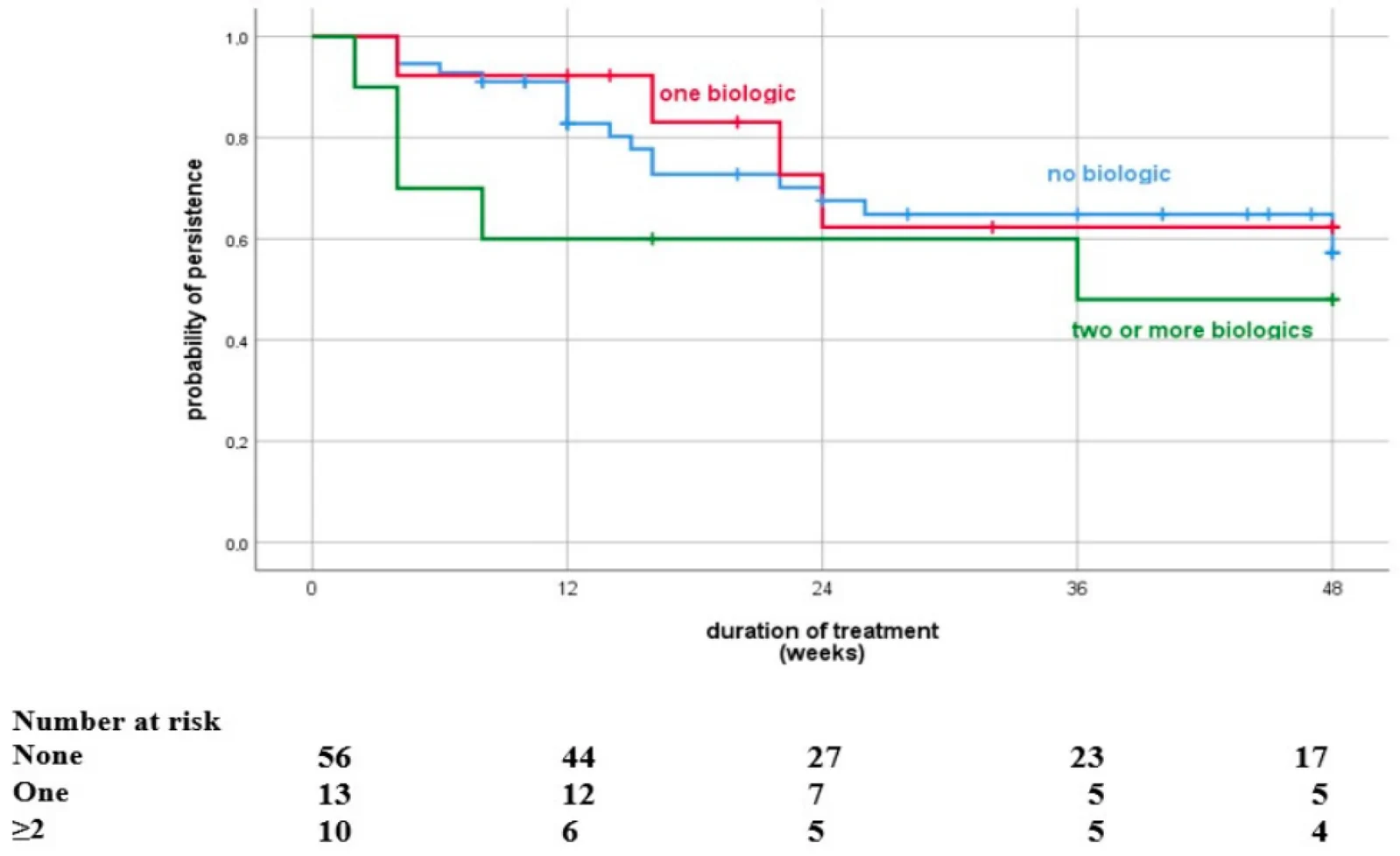
Kaplan–Meier analysis of ozanimod treatment persistence according to the number of prior biologic therapies during the 48-week follow-up period. Patients were stratified as biologic-naïve (n = 56), previously exposed to one biologic therapy (n = 13), or previously exposed to two or more biologic therapies (n = 10). Survival distributions were compared using the log-rank test (*p* = 0.538). Censored observations are indicated by tick marks. Numbers at risk are shown below the plot.

Patients were stratified according to prior biologic exposure into three groups: biologic-naïve, exposure to one biologic therapy, and exposure to two or more biologic therapies, and treatment persistence was analyzed across these groups. Treatment persistence did not differ significantly, according to the number of prior biologic therapies, over the 48-week follow-up period (log-rank *p* = 0.538). Kaplan–Meier estimates of treatment persistence at weeks 12 and 48 were 82.8% and 57.2% in biologic-naïve patients, 92.3% and 62.3% in patients previously exposed to one biologic therapy, and 60.0% and 48.0% in those previously exposed to two or more biologic therapies, respectively (Figure 4).

### 3.8. Impact of Prior Vedolizumab Exposure

**Figure 5:**
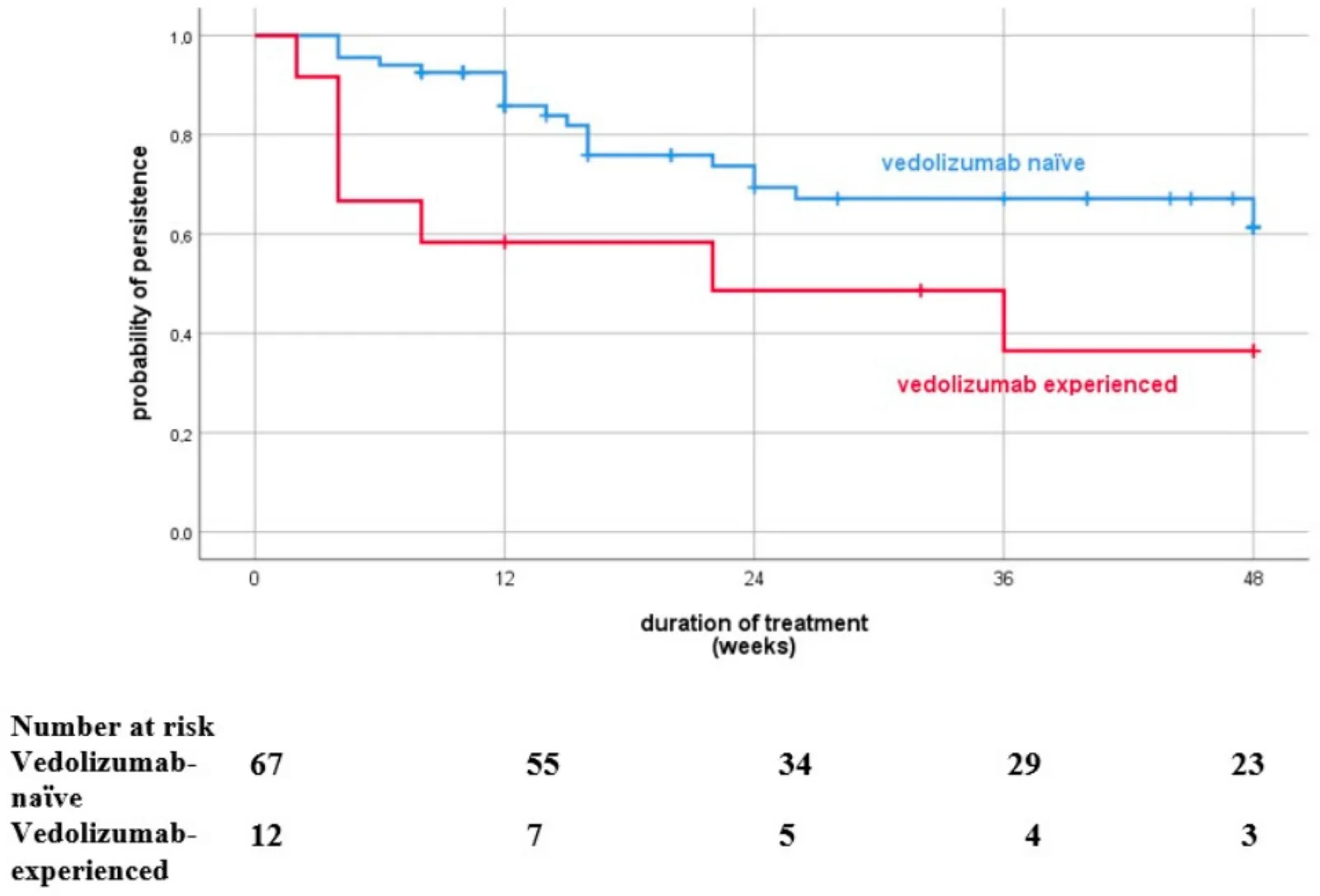
Kaplan–Meier analysis of ozanimod treatment persistence according to previous vedolizumab exposure during the 48-week follow-up period. Patients without previous vedolizumab exposure (n = 67) and those previously exposed to vedolizumab (n = 12) were compared using the log-rank test (*p* = 0.037). Censored observations are indicated by tick marks. Numbers at risk are shown below the plot.

Treatment persistence was significantly lower among patients with previous vedolizumab exposure over the 48-week follow-up period (long-rank *p* = 0.037). Kaplan–Meier estimates of treatment persistence at weeks 12 and 48 were 85.8% and 61.3%, respectively, in patients without previous vedolizumab exposure and 58.3% and 36.5%, respectively, in patients previously exposed to vedolizumab (Figure 5). In univariable Cox regression analyses over the 48-week follow-up period, prior vedolizumab exposure was associated with an increased hazard of treatment discontinuation (HR 2.390, 95% CI 1.010–5.659; *p* = 0.048). Age (0.980, 95% CI 0.956–1.005; *p* = 0.111), sex (male vs. female: HR 0.986, 95% CI 0.457–2.128; *p* = 0.972), corticosteroid use at ozanimod initiation (HR 1.751, 95% CI 0.818–3.748; *p* = 0.149), disease extent (E3 vs. E1/E2: HR 0.913, 95% CI 0.422–1.976; *p* = 0.818), disease duration (HR 0.965, 95% CI 0.911–1.021; *p* = 0.213), and baseline CRP (HR 1.216, 95% CI 0.820–1.803; *p* = 0.331) were not significantly associated with treatment discontinuation. The baseline CRP analysis included 70 patients because of missing baseline CRP values in nine patients.

Given the limited number of treatment-discontinuation events, the multivariable Cox model included prior vedolizumab exposure, age, sex, and corticosteroid use at ozanimod initiation as clinically relevant covariates. The analysis included all 79 patients and 27 treatment-discontinuation events. After adjustment, prior vedolizumab exposure remained associated with an increased hazard of treatment discontinuation (adjusted HR 3.070, 95% CI 1.228–7.672; *p* = 0.016). Age was also associated with a lower hazard of treatment discontinuation (adjusted HR 0.970, 95% CI 0.944–0.997; *p* = 0.030), whereas sex and corticosteroid use at ozanimod initiation were not significantly associated with treatment discontinuation (Supplementary Table S3). The corresponding adjusted Cox-model-derived survival curves are presented separately in Supplementary Figure S1.

Given the clinical relevance of week 12 as an assessment time point, a week-12 Cox regression analysis was performed as an exploratory supplementary analysis of early treatment discontinuation (Supplementary Table S4).

## 4. Discussion

This real-world study showed that ozanimod is associated with satisfactory efficacy and a good safety profile in patients with moderately active UC. Clinical response and remission rates were attained in 77.4% and 71.0% of patients, respectively, while endoscopic improvement was demonstrated in 65.6% of patients at week 12. Furthermore, patients with long-term follow-up had sustained clinical and endoscopic results through week 48. However, the durability of response to ozanimod should be viewed with caution, due to the insufficient number of patients with available week 48 data. This is a downside that reflects the real-world nature of the study, as some patients had not yet reached long-term follow-up at the time of the analysis. Treatment discontinuation and loss to follow-up additionally contributed to missing long-term data.

Efficacy data in our cohort are better than the results of the pivotal phase 3 True North trial, in which clinical response and remission during induction were seen in 47.8% and 18.4% of patients, respectively [7]. This may be partially explained by differences between clinical trial and real-world populations. Patients enrolled in clinical programs are often treated more individually, including optimization of concomitant medicines and tighter follow-up. Moreover, the majority of patients in our study had moderate disease activity, which may have led to better treatment results. The analysis of the regulatory study data showed that ozanimod performs faster and better when the disease burden is low [7].

Our study also showed biochemical improvement with a significant drop in CRP levels in participants with an elevated measurement at baseline. Normalization of CRP, albeit less useful in UC, is nonetheless important and may be associated with superior long-term outcomes and mucosal healing [11]. The CRP decrease in our cohort further signifies the anti-inflammatory impact of ozanimod.

Treatment persistence is an essential surrogate indicator for therapeutic efficacy and safety. In our population, more than half of the patients were still on treatment at any given time point during follow-up. Termination of treatment was mainly due to primary non-response or subsequent loss of response, rather than emergence of adverse effects. Similar persistence rates have been reported for other advanced therapies in UC, including vedolizumab, ustekinumab and JAK inhibitors [12,13].

Treatment persistence did not differ significantly between biologic-naïve and biologic-experienced patients in the overall comparison. Biologic-experienced patients are generally a more difficult -to- treat population, with higher inflammatory burden and treatment failure rates; however, we did not find any statistically significant differences in treatment persistence between biologic-naïve and biologic-experienced patients. Similarly, treatment persistence did not differ significantly when patients were stratified according to the number of prior biologic therapies (0, 1, or ≥2), suggesting that overall prior biologic treatment burden was not significantly associated with persistence in this cohort. These findings are of high practical relevance in light of the rising number of patients who have been exposed to advanced treatments. Similar findings have been reported in the post hoc analysis of the True North program, as well as in real-world cohorts [10,14,15].

Interestingly, previous vedolizumab exposure was associated with lower treatment persistence over the 48-week follow-up period. This finding was supported by both the Kaplan–Meier analysis and Cox regression, with prior vedolizumab exposure persistently associated with an increased hazard of treatment discontinuation after adjustment for age, sex, and corticosteroid use at ozanimod initiation. An exploratory analysis restricted to the first 12 weeks showed a similar association between prior vedolizumab exposure and early treatment discontinuation, although this analysis should be interpreted cautiously given the limited number of early events. Several explanations may account for the association between previous vedolizumab exposure and reduced treatment persistence. Most likely, prior vedolizumab exposure reflects a population with an initially moderately active disease, who later developed a more severe phenotype [16]. Alternatively, our results may indicate a poor response of these patients to the ‘leukocyte trafficking pathway’. Both vedolizumab and ozanimod block lymphocyte trafficking, albeit through different mechanisms, and it is reasonable to assume that failure of one trafficking-targeted therapy may predict lower efficacy of another medication with a similar target. However, safe conclusions cannot be drawn, due to the small number of vedolizumab-exposed patients in the current study. Therefore, this association should be considered exploratory and hypothesis-generating, rather than evidence of a definitive predictive effect and requires confirmation in larger cohorts.

Ozanimod seems to be a reliable therapeutic option for non-severe cases, with a low implementation rate in clinical practice. The positioning of ozanimod in UC treatment has not been established yet. According to our findings, overall previous biologic exposure and the number of prior biologic therapies were not significantly associated with treatment persistence, whereas previous vedolizumab exposure was associated with a higher risk of treatment discontinuation. However, given the small sample size and potential for residual confounding, our findings cannot justify any recommendation regarding ozanimod’s positioning in the therapeutic algorithm.

The safety profile found was generally encouraging and consistent with previously reported registration and post-marketing data [5,17]. Most adverse events were mild or moderate, and termination of treatment because of toxicity was uncommon. The most common laboratory abnormalities were lymphopenia and abnormal transaminases, which are both mechanistically expected with S1P receptor modulation. Importantly, a single patient had to discontinue treatment due to severe lymphopenia. One patient suffered from pulmonary embolism, and a causal link with ozanimod was doubtful. Overall, no unanticipated safety signs were observed during follow-up.

Our study is one of the few that provide data about ozanimod’s efficacy in the real-world context. Patients from many tertiary hospitals were included, thus reliably representing real-world clinical practice in Greece. The enrollment of individuals with previous biologic exposure, concomitant corticosteroid medication, and comorbidities such as multiple sclerosis is also considerable. The study of patients with multiple sclerosis is of special clinical relevance, considering the dual therapeutic effect of ozanimod across both indications. However, MS-specific clinical and neurological outcome data were not available; therefore, the effect of ozanimod in this specific group of patients could not be evaluated and requires further investigation. There are additional, significant limitations to acknowledge. The retrospective observational approach is inherently susceptible to selection bias, poor data collection and unmeasured confounding factors. Strict assessment windows could not be applied, resulting in variability in the timing of baseline, week 12, and week 48 evaluations. Moreover, the relatively small sample size, the limited number of treatment-discontinuation events, and the low number of patients with previous vedolizumab exposure reduce the statistical power and precision of the Cox regression estimates. Although clinically relevant variables were evaluated and the multivariable model was kept parsimonious to reduce the risk of overfitting, residual confounding cannot be excluded. In particular, previous vedolizumab exposure may represent a marker of a more refractory disease course and previous treatment failure. Therefore, findings regarding factors associated with treatment persistence should be considered exploratory and hypothesis-generating and need confirmation in larger prospective cohorts.

## 5. Conclusions

This multicenter, real-world study demonstrated that ozanimod is an effective and well-tolerated therapeutic option for patients with moderately active UC, in routine clinical practice. Good rates of clinical and endoscopic response were observed. Prior vedolizumab exposure was associated with reduced treatment persistence, although this finding requires further investigation in wider cohorts. Prospective studies with larger patient populations, longer follow-up and strict endoscopic endpoints are warranted to further define the positioning of ozanimod within the evolving therapeutic landscape of UC.

## Figures and Tables

**Table 1:** Basic patient characteristics.

**Disease characteristics** E1 6 (7.6%) E2 35 (44.3%) E3 38 (48.1%) Extraintestinal manifestations 5 (6.3%)
**Previous conventional treatment exposure** Oral mesalazine 71 (89.9%) Topical mesalazine 69 (87.3%) Oral corticosteroids 51 (64.6%) Topical corticosteroids 18 (22.8%) Budesonide 25 (31.6%) Azathioprine 22 (27.8%) Methotrexate 2 (2.5%)
**Previous biologic treatment exposure** Biologic-experienced 23 (29.1%) Biologic-naïve 56 (70.9%) One biologic 13 (16.5%) ≥2 biologic therapies 10 (12.7%)
**Type of previous biologic treatment exposure** Vedolizumab 12 (15.2%) Ustekinumab 4 (5.1%) Infliximab 12 (15.2%) Adalimumab 5 (6.3%) Golimumab 3 (3.8%) Tofacitinib 4 (5.1%) Upadacitinib 1 (1.3%)
**Treatment at ozanimod initiation** Steroid use at ozanimod initiation 33 (41.8%)
Laboratory and clinical indices (mean ± SD) or median (IQR) Hemoglobin, (g/dL) 13.23 ± 1.54 Albumin, (g/dL) 4.15 (3.9–4.5) Mayo endoscopic sub-score 2 (2–2) Partial Mayo score5 (3–6) Modified Mayo score 5 (4–6)

Data are presented as number (%) or mean ± standard deviation or median (interquartile range), as appropriate.

**Table 2:** Adverse events during ozanimod treatment.

Adverse Event	n
**Serious adverse events (SAE)**	
Pulmonary embolism	1
Immune thrombocytopenia (ITP)	1
**Treatment discontinuation due to adverse events**	
Transaminasemia	1
Severe lymphopenia	1
**Adverse events (AE)**	
Lymphopenia (no discontinuation)	3
Transaminasemia	3
Herpetic infection	1

## Data Availability

Data regarding the study, reported results and analyzed datasets can be found at “Department of Gastroenterology, “Alexandra” General Hospital of Athens, Athens”.

## Supplementary Materials

The following supporting information can be downloaded at: https://www.mdpi.com/article/10.3390/medicina62091711/s1. Supplementary Table S1. Comparison of baseline characteristics between patients with and without available follow-up data at weeks 12 and 48. Supplementary Table S2. Reasons for ozanimod discontinuation according to prior biologic exposure. Supplementary Table S3. Impact of Prior Vedolizumab Exposure on Treatment Discontinuation During the 48-Week Follow-up. Supplementary Table S4. Impact of Prior Vedolizumab Exposure at week 12. Supplementary Figure S1. Adjusted Cox-model-derived survival curves for treatment persistence according to prior vedolizumab exposure. Predicted treatment persistence over the 48-week follow-up period is shown for vedolizumab-naïve and vedolizumab-experienced patients. Curves were derived from a multivariable Cox proportional hazards model adjusted for age, sex, and corticosteroid use at ozanimod initiation. The analysis included 79 patients and 27 treatment-discontinuation events.

## Abbreviations

The following abbreviations are used in this manuscript:

UC	ulcerative colitis
IBD	inflammatory bowel disease
TNF	tumor necrosis factor-alpha
JAK	Janus kinase
S1P	sphingosine-1-phosphate
FDA	Food and Drug Administration
EMA	European Medicines Agency
SD	standard deviation
IQR	interquartile range
