# Supplementary material for: Efficacy and Safety of Ozanimod in Ulcerative Colitis: A Real-World Pan-Hellenic Study
Source: Medicina (Kaunas). 2026 Sep 6;62(9):1711. doi: 10.3390/medicina62091711 (PMC13609869; doi:10.3390/medicina62091711)
Supplement: Supplementary file 1 [file medicina-62-01711-s001.zip › medicina-4452689-supplementary.pdf]

Supplementary Table S1. Comparison of baseline characteristics between patients with and without available follow-up data at weeks 12 and 48

Baseline characteristic	Follow-up data available	Follow-up data unavailable	p-value	Effect size (r)
**Week 12**	**n=62**	**n=17**		
**Age, years, median (IQR)**	45.5 (33.5–57.3)	32.0 (25.0–50.5)	0.060	0.21
**Baseline partial Mayo score, median (IQR)**	4 (3–5)	4 (3–5)	0.540	0.07
**Baseline Mayo endoscopic sub-score, median (IQR)**	2 (2–2)	2 (2–3)	0.074	0.20
**Baseline CRP, mg/dL, median (IQR)**	0.37 (0.10–1.19), n=56	0.40 (0.15–1.23), n=14	0.808	0.03
**Previous biologic exposure, n (%)**	18/62 (29.0%)	5/17 (29.4%)	0.976	—
**Corticosteroid use at ozanimod initiation, n (%)**	25/62 (40.3%)	8/17 (47.1%)	0.618	—
**Week 48**	**n=26**	**n=53**		
**Age, years, median (IQR)**	48.5 (37.3–58.5)	41.0 (27.5–53.5)	0.100	0.18
**Baseline partial Mayo score, median (IQR)**	4 (3–4.25)	4 (3–5)	0.440	0.09
**Baseline Mayo endoscopic sub-score, median (IQR)**	2 (2–2)	2 (2–2)	0.340	0.11
**Baseline CRP, mg/dL, median (IQR)**	0.34 (0.09–1.14), n=22	0.40 (0.12–1.20), n=48	0.420	0.10
**Previous biologic exposure, n (%)**	8/26 (30.8%)	15/53 (28.3%)	0.821	—
**Corticosteroid use at ozanimod initiation, n (%)**	13/26 (50.0%)	20/53 (37.7%)	0.299	—

Data are presented as median (IQR) or n (%), as appropriate. Baseline CRP was available for 70/79 patients (88.6%); therefore, comparisons involving CRP were restricted to patients with available baseline CRP measurements. Continuous variables were compared using the Mann–Whitney U test and categorical variables using the chi-square test. Effect sizes for continuous variables were calculated as r = |Z|/√N; effect sizes were not calculated for categorical comparisons.

No statistically significant differences in age, baseline partial Mayo score or baseline, Mayo endoscopic sub-score were observed between patients with and without available follow-up data at weeks 12 and 48. Baseline CRP data were available for 70/79 patients (88.6%). Among patients with available week-12 follow-up data (n=56), median baseline CRP was 0.37 mg/dL (IQR 0.10–1.19), compared with 0.40 mg/dL (IQR 0.15–1.23) among those without available week-12 follow-up data (n=14), with no significant difference between groups (p=0.808).

Among patients with available baseline CRP data, median CRP did not differ significantly between those with (n=22) and without (n=48) available week-48 follow-up data [0.34 mg/dL (IQR 0.09–1.14) vs. 0.40 mg/dL (IQR 0.12–1.20), respectively; p=0.420]. Data on corticosteroid use at ozanimod initiation were available for all 79 patients. Corticosteroid use did not differ significantly between patients with and without available follow-up data at week 12 [25/62 (40.3%) vs. 8/17 (47.1%), respectively; p=0.618] or week 48 [13/26 (50.0%) vs. 20/53 (37.7%), respectively; p=0.299]. Previous biologic exposure did not differ significantly between patients with and without available follow-up data at week 12 [18/62 (29.0%) vs. 5/17 (29.4%), respectively; p=0.976] or week 48 [8/26 (30.8%) vs. 15/53 (28.3%), respectively; p=0.821]

Supplementary Table S2.
Reasons for ozanimod discontinuation according to prior biologic exposure

Treatment-history group	Primary non-response	Secondary loss of response	Adverse event	Total discontinuations
Biologic-naïve	8	8	2	18
Biologic-experienced	6	2	2	10

Supplementary Figure S1

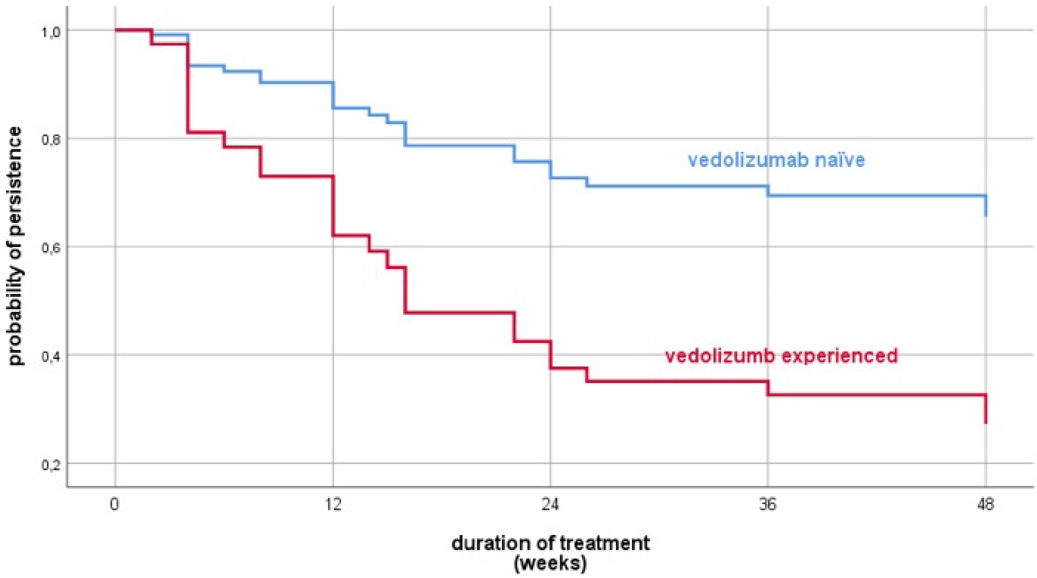

Supplementary Figure S1. Adjusted Cox-model-derived survival curves for treatment persistence according to prior vedolizumab exposure. Predicted treatment persistence over the 48-week follow-up period is shown for vedolizumab-naïve and vedolizumab-experienced patients. Curves were derived from a multivariable Cox proportional hazards model adjusted for age, sex, and corticosteroid use at ozanimod initiation. The analysis included 79 patients and 27 treatment-discontinuation events.

Supplementary Table S3.
Impact of Prior Vedolizumab Exposure on Treatment Discontinuation During the 48-Week Follow-up

Variable	Unadjusted HR (95% CI)	p-value	Adjusted HR (95% CI)	p-value
**Age, per year**	0.980 (0.956–1.005)	0.111	0.970 (0.944–0.997)	0.030
**Male vs female**	0.986 (0.457–2.128)	0.972	0.905 (0.413–1.983)	0.803
**Prior vedolizumab exposure**	2.390 (1.010–5.659)	0.048	3.070 (1.228–7.672)	0.016
**Corticosteroid use at ozanimod initiation**	1.751 (0.818–3.748)	0.149	1.839 (0.849–3.983)	0.122
**Disease extent, E3 vs E1/E2**	0.913 (0.422–1.976)	0.818	—	—
**Disease duration, per year**	0.965 (0.911–1.021)	0.213	—	—
**Baseline CRP, per mg/dL***	1.216 (0.820–1.803)	0.331	—	—

**HR, hazard ratio; CI, confidence interval; CRP, C-reactive protein. Reference categories were female sex, no previous vedolizumab exposure, no corticosteroid use at ozanimod initiation, and E1/E2 disease extent. The multivariable Cox proportional hazards model included age, sex, previous vedolizumab exposure, and corticosteroid use at ozanimod initiation. The multivariable analysis included 79 patients and 27 treatment-discontinuation events. Baseline CRP was available for 70 patients.*

Supplementary Table S4.

Impact of Prior Vedolizumab Exposure at week 12

	**Univariate analysis**		**Multivariate analysis**	
	HR (95% CI)	*p-value*	Adjusted HR (95% CI)	*p-value*
**Age**	0.970 (0.935-1.007)	0.108	0.955 (0.917-0.995)	0.028
**Sex**	1.682 (0.527-5.365)	0.380	0.727 (0.214-2.466)	0.609
**Vedolizumab exposure**	3.759 (1.255-11.258)	0.018	4.120 (1.264 – 13.425)	0.019
**Steroids at onset**	3.619 (1.134-11.549)	0.030	4.031 (1.223-13.287)	0.022
**Disease extent (E1 and E2, E3)**	1.076 (0.377-3.067)	0.892	-	
**Disease duration**	0.953 (0.880-1.031)	0.232	-	
**CRP at baseline**	1.160 (0.734-1.833)	0.526	-	

In univariable Cox regression analysis, previous vedolizumab exposure (HR 3.75, 95% CI 1.25–11.25; p=0.018) and corticosteroid use at ozanimod initiation (HR 3.61, 95% CI 1.13–11.54; p=0.030) were significantly associated with treatment discontinuation. In contrast, disease extent (E1 and E2, E3), disease duration, baseline CRP, age, and sex were not significantly associated with treatment discontinuation in univariable analyses. Given the limited number of discontinuation events, the multivariable model was kept parsimonious and included previous vedolizumab exposure, corticosteroid use at ozanimod initiation, age, and sex. In the multivariable analysis, previous vedolizumab exposure (adjusted HR 4.12, 95% CI 1.26–13.42; p=0.019) and corticosteroid use at ozanimod initiation (adjusted HR 4.03, 95% CI 1.22–13.28; p=0.022) remained significantly associated with treatment discontinuation. Age was also significantly associated with treatment discontinuation (adjusted HR 0.95, 95% CI 0.91–0.99; p=0.028), whereas sex was not (p=0.609).
